# The impact of influences in a medical screening programme invitation: a randomized controlled trial

**DOI:** 10.1093/eurpub/ckad067

**Published:** 2023-05-02

**Authors:** Christian Patrick Jauernik, Or Joseph Rahbek, Thomas Ploug, Volkert Siersma, John Brandt Brodersen

**Affiliations:** Department of Public Health, The Research Unit for General Practice and Section of General Practice, University of Copenhagen, Copenhagen, Denmark; The Primary Health Care Research Unit, Zealand Region, Sorø, Denmark; Department of Public Health, The Research Unit for General Practice and Section of General Practice, University of Copenhagen, Copenhagen, Denmark; The Primary Health Care Research Unit, Zealand Region, Sorø, Denmark; Centre for Applied Ethics and Philosophy of Science, Department of Communication and Psychology, Aalborg University Copenhagen, Copenhagen, Denmark; Department of Public Health, The Research Unit for General Practice and Section of General Practice, University of Copenhagen, Copenhagen, Denmark; Department of Public Health, The Research Unit for General Practice and Section of General Practice, University of Copenhagen, Copenhagen, Denmark; The Primary Health Care Research Unit, Zealand Region, Sorø, Denmark

## Abstract

**Background:**

Invitations to screening programmes may include influences that are intending to increase the participation rates. This study had two objectives: (i) to assess if different categories of influences had a significant effect on the intention to participate in a screening programme for a fictitious disease and (ii) whether participants were aware of the influences, and if the intention to participate was associated to this awareness.

**Methods:**

A seven-armed randomized controlled trial. Six hundred passers-by were randomly allocated to receive one of seven pamphlets inviting to a fictitious screening programme (neutral, relative risk reductions, misrepresentation of harms, pre-booked appointment, recommendation of participation, fear appeals, all combined). Participants were surveyed to assess (i) intention to participate (ITP) in the screening programme and (ii) awareness of an exerted influence. Chi-squared test was used to calculate the effect of the influences on ITP and the association of ITP with indicating awareness of an exerted influence and correctly locating an influence.

**Results:**

Five hundred and eighty-nine participants were included for analysis. ITP was significantly increased (*P* < 0.05) in three pamphlets (misrepresentation of harms, fear appeals, all combined) [adjusted odds ratio (OR) 4.84, 95% confidence interval (CI): 2.54–9.23; OR 2.45, 95% CI: 1.31–4.59; OR 9.02, 95% CI: 4.44–18.34]. A percentage of 60.0–78.3 participants did not indicate awareness. Awareness was associated with a decreased ITP for those who could locate the influence (OR 0.39, 95% CI: 0.21–0.72) and those who failed to locate the influence (OR 0.47, 95% CI: 0.30–0.74).

**Conclusion:**

The application of influences should be carefully considered for interventions where an informed choice is desired.

## Introduction

Screening programmes for different cancers are implemented in many developed countries. They have intended benefits including reduction in mortality and morbidity plus less radical treatments.^1^ However, cancer screening programmes come with many unintended harms such as false-positive results, overdiagnosis and overtreatment, possibly leading to physical, psychological or social harms.^2^ The quality of screening programmes is sometimes evaluated by a considerable participation rate.^3–5^ From a healthcare authority perspective, if a screening programme for cancer diseases is assessed to do more good than harm, then a high participation rate would maximize the assessed benefit of that screening programme. Furthermore, citizens who are of a lower socioeconomic status have a higher cancer disease incidence (except for breast cancer) but are less likely to participate in the screening programmes.^6–8^ This creates another incentive for health authorities to make screening participation barrier-free and simple in order to promote equality in health. The healthcare authorities can systematically influence citizens in subtle ways that may increase participation rates without making the choice to participate adequately informed. These influences are sometimes referred to as ‘nudging’.^9^ To avoid any misconceptions, we use the broader term ‘influences’.

It is understandable that health authorities would actively use influences to maximize potential benefits of cancer screening programmes, but by defining participation as ‘the right choice’ for all, the health authorities’ interest conflicts with the interest of the individual citizens. Not all citizens will share the same assessment of the benefits/harms as the health authorities. And even if they agree with the health authorities that the benefits outweigh the harms on a population level, they may still not wish to participate because they on an individual level might receive more harm than benefit—current evidence suggests that the more informed citizens are less likely to participate in cancer screening.^10^^,^^11^

A scoping review from 2019 identified influences intended to increase participation that appeared in invitations to cancer screening programmes and constructed five categories of these influences^12^: (i) tendentious presentation of statistics, (ii) omission of harms/emphasis on benefits, (iii) recommendations of participation, (iv) opt-out systems and (v) Fear appeals. If these types of influences significantly affect individual participation by virtue of bypassing or thwarting reflection, they may be incompatible with informed decision-making.^13^^,^^14^

Previous studies have found a considerable increase in both the intention to participate in screening programmes (relative risk reductions^15^) and actual participation (pre-booked appointments^16^), but different study designs prevent comparison between the effects of said influences. Therefore, the primary aim of this study was to evaluate whether these five categories of influences had a significant effect on citizens’ intention to participate in a fictitious medical screening programme. A secondary aim was to evaluate whether citizens were aware of an effort to influence their choice, and if this awareness was associated with the intention to participate.

## Methods

The study followed a seven-arm randomized controlled design that took place in 19 different public locations (libraries, citizen service centres, parks and town squares) in Copenhagen and six proximal municipalities through May–July 2017.

### Participants

Passers-by assessed by the interviewers to be above the age of 18 were selectively approached to attain a wide distribution of age and sex. The number and sex of passers-by who refused to participate or did not respond were recorded. Sociodemographic data (age, sex, level of education and employment status) for every 10th non-participant was collected. If the 10th non-participant refused to answer these questions, the following non-participant was asked until data was obtained.

Potential participants were not included if they were illiterate, had insufficient Danish language skills or were too busy to commit the necessary time (at least 5 minutes) for the interview.

### Material

The authors created an invitational pamphlet (four A5 sheets with a total of 606 words) in Danish that invited the receiver to attend a medical screening programme for a fictitious, non-communicable life-threatening disease named ‘cytoliosis’. It was anticipated that the threat of cancer would trigger particularly strong emotions in respondents, and thus lead to cancer-specific results. Therefore, cytoliosis was invented to eliminate any preconceptions and fears regarding cancer.

The pamphlet for screening for cytoliosis was partially based on the Danish colorectal cancer (CRC) screening pamphlet, and cytoliosis shared the same incidence and mortality as CRC.^17^ The screening programme for cytoliosis shared the same benefits (e.g. mortality reduction) and harms (e.g. false-positives, physical harm and overtreatment) as CRC screening for a 50–60-year-old male. The harms of the fictitious screening programme were increased compared with CRC screening to better balance the benefits and harms of participation.

Five other pamphlets were made based on the ‘neutral’ one (A). Each of these incorporated one of the categories of influences mentioned in the introduction. One pamphlet made use of relative risk reductions (B) to emphasize mortality reduction. A third pamphlet omitted harms and emphasized benefits (C). Pre-booked appointments (D) were introduced in a fourth pamphlet as an example of an opt-out system and the fifth pamphlet contained explicit recommendation for participation (E) from the Danish Health Agency and Danish Patient Society. A sixth pamphlet used fear appeals (F), e.g. underlining the severity of the disease. Finally, a pamphlet combining all five influences (G) was made. See Supplementary appendix SA for all the translated pamphlets.

All the types of influence studied were inspired by actual examples from cancer screening programmes.^12^

One hundred pilot interviews were conducted to evaluate face validity, content validity and project feasibility and to develop an interview guide for the present study.

### Sample size determination

An *a priori* power analysis determined a sample size of minimum of 69 participants per pamphlet to give 80% power to detect a 20-percentage point difference of intention to participate between the group receiving a neutral pamphlet (A) and the groups receiving a pamphlet containing an influence (B–G). To achieve greater statistical certainty, the number of interviews for the neutral pamphlet (A) was doubled, totalling the number of participants across all pamphlets to a minimum of 552, why the authors decided on 600 interviews in total, to allow for potential dropouts and exclusions.

### Randomization and interviews

Each pamphlet was assigned according to a randomized, computer-generated allocation sequence where the neutral pamphlet (A) occurred twice as much as each of the other pamphlets [2; 1; 1; 1; 1; 1; 1]. Recruitment and the structured interviews were based on an interview guide (see Supplementary appendix SB). One of two interviewers would conduct the interview, the other would code the answers and recruit participants. When a passer-by consented to participate, only the interviewer conducting the interview would look at a printed version of the allocation sequence and assign the participant the corresponding pamphlet. It was explained to the participant that cytoliosis is a fictitious, non-communicable life-threatening disease and was required to read the whole pamphlet before answering questions according to the interview guide. None of the participants knew that they took part in a multi-arm randomized controlled trial with alternative pamphlets to their assigned one. Each interview lasted 5–10 minutes.

### Primary outcome: intention to participate

The effect of the influences was measured by differences in intention to participate between the neutral pamphlet (A) and the pamphlets containing the influences (B–G). Intention to participate was measured by asking the surveyed person if they wanted to participate in the screening programme *if* cytoliosis was a real disease. To pursue a dichotomous outcome, the surveyed person was required to answer either yes or no.

### Secondary outcomes

To evaluate whether the surveyed persons were aware of the effort to influence their choice each surveyed person was asked which of two statements he/she agreed with the most: ‘The pamphlet provides me information in order to help me make my own choice’ or ‘The pamphlet provides me information in order to direct me towards the choice that the pamphlet favours’. If the surveyed person felt that the pamphlet tried to direct their choice, (s)he was asked if there were anywhere specific in the pamphlet that made them feel so. It was then noted if the selected paragraph contained an influence or not. More general responses were also recorded, i.e. ‘the first page is trying to scare me’ or ‘there is no mention of harms’. It was then decided by the coding interviewer whether the response indicated awareness or non-awareness to the influence.

The effect of awareness of the influences on intention to participate was assessed by comparing odds ratios (ORs) of intention to participate between three groups: one group who felt the pamphlet tried to help the receiver make their own choice, one group who felt the pamphlet tried to direct their choice and subsequently correctly located an influence and the last group who felt the pamphlet was trying to direct their choice but failed to correctly locate an influence.

### Sociodemographic

Information regarding age, sex, level of education, employment status, household status and mother tongue was obtained through a simple, self-reported questionnaire following each interview.

### Statistical analysis

Chi-squared (χ^2^) tests were used to assess both the relation between the influences and the intention to participate (primary outcome) as well as the effect of the awareness of the influences had on the intention to participate (secondary outcome).

For the primary outcome, ORs from logistic regression analysis were used to assess the difference in intention to participate for each pamphlet relative to the neutral pamphlet (A), both unadjusted and adjusted for all the pre-determined gathered sociodemographic data including the location of recruitment. For analysis, age was grouped into four categories: under 30, 31–45, 46–60 and over 60. For the secondary outcome, logistic regression analysis was used to obtain ORs and adjust for the distribution of the pamphlets in each group.

The statistician was blinded to the group allocations during the whole process.

A *P* value < 0.05 was deemed statistically significant.

### Contribution

All listed authors attest that they meet authorship criteria and that no others meeting the criteria have been omitted. The study design was conceived by all authors. O.J.R. and C.P.J. drafted the interview guide and the pamphlets, and T.P. and J.B. provided comments that led to revisions. All interviews were conducted and coded by O.J.R. and C.P.J. V.S. generated the allocation sequence and conducted statistical analyses. O.J.R. and C.P.J. drafted the manuscripts and T.P., J.B. and V.S. contributed to revisions. O.J.R. and C.P.J. are guarantors for this paper.

### Ethics issues

According to the Danish Scientific Ethical Committees Act §1 stk. 4, this project does not require review from the committee, as it is not classified as a health research study. No personal data were collected. All participants were informed before participation that cytoliosis and the screening programme were fictitious.

## Results

### Participant characteristics

The authors approached 1489 passers-by (718 men, 771 women). Of those, 600 committed to participate (40.30%), where two were excluded due to age under 18 (one from group B and one from group D). Nine dropped out of the study while reading the pamphlet but before the interview: eight because they could not provide the necessary time (two from group A, four from group B, one from group E and one from group F) and one because of insufficient Danish skills (one from group B). This left 589 for analysis. See the flowchart in Supplementary appendix SC.

Participants differed from the sampled non-participants: Participants were generally longer educated, younger and more often employed or studying. See table 1 for participant and non-participant characteristics and Supplementary appendix SD for sociodemographic data stratified across pamphlets. Missing replies: not all participants completed the sociodemographic questionnaire.

**Table 1:** Participant and non-participant sociodemographic data

	Participants	Sampled non-participants	*P*-value (difference)
	*n* (%)	*n* (%)	
Total	589	89	
Age			0.4897
≤30 years old	124 (21.2)	15 (16.9)	
31–45 years old	180 (30.8)	26 (29.2)	
46–60 years old	150 (25.7)	22 (24.7)	
>60 years old	130 (22.3)	26 (29.2)	
Missing replies: age	5 (NA)	0 (NA)	
Sex			0.5267
Male	252 (43.0)	41 (46.6)	
Female	334 (57.0)	47 (53.4)	
Missing replies: sex	3 (NA)	1 (NA)	
Education			0.0002
Primary and lower secondary education	35 (5.9)	18 (20.2)	
Higher secondary education	63 (10.7)	13 (14.6)	
Vocational education	50 (8.5)	7 (7.9)	
Short-cycle higher education	50 (8.5)	7 (7.9)	
Medium-cycle higher education	187 (31.8)	22 (24.7)	
Long-cycle higher education	204 (34.6)	22 (24.7)	
Missing replies: education	0 (NA)	0 (NA)	
Employment status			0.0124
In employment	330 (56.0)	38 (42.7)	
Studying	82 (13.9)	9 (10.1)	
Unemployed	67 (11.4)	18 (20.2)	
Retired	110 (18.7)	24 (27.0)	
Missing replies: employment status	0 (NA)	0 (NA)	
Household status			
Living alone	227 (38.5)	Not obtained	
Living together	362 (61.5)	Not obtained	
Missing replies: household status	0 (NA)	NA	
Mother tongue			
Danish	525 (89.3)	Not obtained	
Other	63 (10.7)	Not obtained	
Missing replies (mother tongue)	1 (NA)	NA	

NA, not applicable.

### Primary outcome: intention to participate across the influences

The lowest proportion of intention to participate (31.8%) was seen in the group receiving the neutral pamphlet (A), while the proportion from the other pamphlets ranged between 39.2% and 80.0%. An OR > 1 was seen across all pamphlets comprising influences both adjusted and unadjusted for all gathered sociodemographic data, including the location of recruitment (table 2). The unadjusted intention to participate was statistically significantly increased in the groups receiving pamphlets containing relative risk reductions (B), misrepresentation of harms vs. benefits (C), explicit recommendation of participation (E), fear appeals (F) and all influences combined (G). When adjusted for sociodemographic status, pamphlets (C), (F) and (G) had statistically significantly increased intention to participation rates.

**Table 2:** The intention to participate for each pamphlet with an influence (B–G) relative to the neutral pamphlet (A)

Pamphlet	Total	Drop out/excluded	Intend	Not intend	Unadjusted for sociodemographic data		Adjusted for sociodemographic data	
	*n* (%)	*n*	*n* (%)	*n* (%)	OR (95% CI)	*P*-value	OR (95% CI)	*P*-value
Neutral (A)	148 (25.1)	2	47 (31.8)	101 (68.2)	Ref	<0.0001	Ref	<0.0001
Relative risk reductions (B)	69 (11.7)	6	34 (49.3)	35 (50.7)	2.09 (1.16*–*3.75)	0.0137	1.87 (0.98*–*3.57)	0.0585
Misrepresentation of harms vs. benefits (C)	75 (12.7)	0	50 (66.7)	25 (33.3)	4.30 (2.38*–*7.77)	<0.0001	4.84 (2.54*–*9.23)	<0.0001
Pre-booked appointments (D)	74 (12.6)	1	29 (39.2)	45 (60.8)	1.38 (0.77*–*2.48)	0.2721	1.15 (0.61*–*2.16)	0.6586
Explicit recommendations (E)	74 (12.6)	1	34 (46.0)	40 (54.0)	1.83 (1.03*–*3.24)	0.0395	1.86 (1.00*–*3.47)	0.0512
Fear appeals (F)	74 (12.6)	1	39 (52.7)	35 (47.3)	2.39 (1.35*–*4.25)	0.0028	2.45 (1.31*–*4.59)	0.0049
Combined (G)	75 (12.7)	0	60 (80.0)	15 (20.0)	8.60 (4.43*–*16.69)	<0.0001	9.02 (4.44*–*18.34)	<0.0001

### Secondary outcome: awareness of the influences

A majority varying from 60.0% to 78.3% did not indicate awareness that their choice was trying to be influenced (pamphlets B–G). There was no clear difference between the answers to the neutral pamphlet (A), and the pamphlets containing a deliberate attempt at influencing the participants choice. The participants who found that the pamphlet tried to direct their choice could correctly locate the influences more often than not in pamphlets C, F and G, while almost none were able to correctly locate the influences in pamphlets B, D and E. See table 3 for details.

**Table 3:** The number of participants indicating awareness of the influences and ability to correctly locate the influences, stratified for each pamphlet

Pamphlet	‘The pamphlet provides me information in order to help me make my own choice’.	‘The pamphlet provides me information in order to direct me towards the choice that the pamphlet favours’.	
		Influences incorrectly located	Influences correctly located
	*n* (%)	*n* (%)	*n* (%)
A	107 (72.3)	41 (27.7)	NA
B	54 (78.3)	12 (17.4)	3 (4.3)
C	46 (61.3)	12 (16.0)	17 (22.7)
D	49 (66.2)	22 (29.7)	3 (4.1)
E	48 (64.9)	20 (27.0)	6 (8.1)
F	52 (70.3)	8 (10.8)	14 (18.9)
G	45 (60.0)	8 (10.7)	22 (29.3)

NA = Not applicable.

### Secondary outcome: the effect of awareness of the influences on intention to participate

Participants receiving a pamphlet with an influence (B–G), who did not indicate awareness that their choice was trying to be influenced had an increased intention to participate compared to both those who felt the pamphlet was trying to direct their choice and subsequently correctly located an influence, and those who felt the pamphlet was trying to direct their choice but failed to correctly locate an influence (table 4). There was no statistically significant difference between the two groups indicating awareness. Stratification of the results for each pamphlet was not conducted, due to the lack of statistical power.

**Table 4:** Intention to participate stratified for the indication of awareness of the influences, adjusted

	‘The pamphlet provides me information in order to help me make my own choice’.	‘The pamphlet provides me information in order to direct me towards the choice that the pamphlet favours’.	
Influences correctly or incorrectly located	Not applicable	Influences incorrectly located	Influences correctly located
		OR (95% CI)	OR (95% CI)
Intention to participate (adjusted for distribution of pamphlets in each group)	1.00 (reference)	0.47 (0.30*–*0.74)	0.39 (0.21*–*0.72)

## Discussion

All five categories of influences proved to increase intention to participate with an adjusted OR > 1. Less than half of the participants indicated awareness of the influences, and not indicating awareness was associated with an increased intention to participate. For those who indicated a general awareness of influences, there was no statistically significant difference in intention to participate between those who correctly and those who incorrectly located the influences.

A strength of this study is the randomized design where blinding of the interviewers was broken only after recruitment and thereby eliminating possible confounding. The interviews were conducted in consistence with a structured guide that addressed potential biases associated with breaking the blinding. Another potential strength is using an unknown, fictitious disease as cytoliosis eliminating fears and preconceptions associated with, e.g., cancer and well-known life-threatening diseases that could overestimate the intention to participate across all pamphlets. Our primary outcome was calculated as intention to participate, which we consider a strength because the intention to participate is regarded as a valid outcome of informed choice regarding cancer screening participation.^11^^,^^18^

A potential limitation of the present study is the risk of an interviewer’s effect on participants who might strive to answer what they believe is confirming the hypothesis of the researchers. This was addressed during the interviews through the structured interview guide.

The results of the secondary outcomes should be interpreted with caution. Since the secondary outcomes are measured after the participants indicate their intention to participate, it may affect their response as to whether the pamphlet was trying to direct their choice. We hypothesize that the participants who intended to participate might be more reluctant to admit that they have been potentially influenced. This would understate the measured awareness in the groups with the highest intention to participate.

Another limitation could be that the external validity of the study is limited due to cytoliosis being a fictitious disease. This might explain why pamphlet (D) (pre-booked appointments) has a relatively low effect size (adjusted OR 1.15) since participants could not relate to opting out of a ‘false’ screening programme. This could explain the discrepancy with a different study that found that the impact of pre-booked appointments compared to mailed reminders almost doubled participation within 6 months (risk ratio 1.9, 95% CI: 1.5–2.3).^16^ Another example of a pamphlet that might underestimate the true effect of an influence is pamphlet B with the use of relative risk reductions. Compared to other studies that looked into the effect of informing absolute risk reductions instead of relative risk reductions our set-up finds a smaller effect on the intention to participate.^15^^,^^19^ The hypothetical scenario of our design removes any real-life factors in the decision-making, and since previous research of the influences shows a greater effect in real life than in our study, we hypnotize that our design underestimates the effect of the influences in real-life settings.

Before and after adjustment all six pamphlets proved an OR >1. We hypothesize that the statistically insignificant ORs might prove significant in a larger population. Regardless, decision-makers should take into consideration that a slightly positive OR can translate to great numbers of influenced participants in screening populations.

Not indicating awareness of being influenced is associated with a stronger intention to participate. Citizens who are less alert to attempts at influencing their choices might therefore enrol in a screening programme because of being influenced rather than being convinced by unbiased information. These results beg consideration and discussion of the use of different types of influences applied to increase the participation rate in cancer screening programmes. The potential harms of participating in cancer screening programmes can be severe and substantial, and the intended effect of increasing the participation rate using influences must be carefully weighed against the unintended effect of potentially bypassing the participants’ informed choice. This indicates a need for alternative ways of appraising cancer screening programmes than by participation rate. One such alternative could be the rate of informed decisions by potential screening participants. However, appraising screening programmes by the rate of informed choice introduces new problems. Citizens might feel distressed trying to comprehend the many consequences, benefits and harms of the offered programmes.^20^^,^^21^ A perception gap appears when lay people are informed about benefits and harms, possibly due to a gross overestimation of benefits and underestimation of the harms.^22^^,^^23^ It may therefore turn out to be difficult to make them pass a threshold of acceptable understanding. Furthermore, this tedious recruitment method can unintendedly influence citizens towards non-participation. But if citizens are offered screening and therefore put in a situation where they must make a choice there may be no better alternative.

For future research, it is relevant to investigate the potential negative effects of using the influences. An example of such a negative effect is weakened trust in healthcare authorities.^14^^,^^24^

Informational material is not the only aspect of decision-making, and this study does not examine external reasons for the participants’ choices, e.g. society’s (health) culture, one’s own and society’s general attitudes to health interventions, sense of duty, behaviour and opinions of relatives, barriers to intention and actual behaviour, health professionals’ financial incentives to increase screening uptake, etc. The impact of informational material on actual choice may be limited.^25–27^ Research studying the external reasons may quantify the importance of decision-making on informational material.

In conclusion, the considerable effect of the influences that are further enhanced by non-awareness suggests that the application of such influences should be carefully considered for interventions where participation on an informed basis is intended. Further research about the potential negative effects of influences is warranted, i.e. weakened trust in health authorities.

## Supplementary Material

ckad067_Supplementary_Data

## Data Availability

The complete dataset is available upon request to the corresponding author.
