# Supplementary material for: The impact of influences in a medical screening programme invitation: a randomized controlled trial
Source: Eur J Public Health. 2023 May 2;33(3):509–14. doi: 10.1093/eurpub/ckad067 (PMC10234657; doi:10.1093/eurpub/ckad067)
Supplement: ckad067_Supplementary_Data [file ckad067_supplementary_data.zip › ckad067_Supplementary_Data/ejph-2022-11-om-0544-File002.pdf]

APPENDIX A – PAMPHLETS TRANSLATED FROM ORIGINAL DANISH

<table>
<tr><td>Pamphlet</td><td>Everything underlined is something added to or altered from pamphlet A. Anything in cursive is something removed from pamphlet A.</td></tr>
<tr><td>Translated content pamphlet A</td><td>An offer of a test for cytoliosis
You can get yourself screened for cytoliosis, which is a non-communicable, non-hereditary disease that can be life-threatening.

What is my risk?
1 out of 800 persons in your age-group develop cytoliosis each year. The remaining 799 persons do not.

Symptoms of the disease are known. The purpose of the test is to catch the disease before the symptoms show.

In this pamphlet, you can get a better understanding of the disease to help you decide whether to accept the offer of the test or not.

By participating in the screening programme you can reduce your risk of dying from the disease, but participation might cause harm (see p. 2 for benefits/harms).

[End of page 1]

Potential benefits of being screened

Lower mortality of the disease
By participating in the screening programme, you can reduce your risk of dying from the disease – 18 out of 10.000 will die from the disease. With this screening programme 14 out of 10.000 will die from the disease. Hence, 4 out of 10.000 will avoid dying of cytoliosis.

Milder treatment options
If the disease is caught early there is a better chance that you can be treated by milder procedures, and your risk of going through pharmaceutical treatment is reduced.

[End of column 1, page 2]
Potential harms of being screened

Worry and false alarm
Even if the initial test shows sign of the disease, it doesn't mean that you have it – waiting for the result and the follow-up examination can lead to worry and unrest. If 10.000 persons participate in 5 consecutive screening rounds in 10 years, 3.000 persons will experience a false alarm at least once.

Physical harm:
Of the 3.000 persons who get a false alarm, 9 will experience physical harm from the follow-up examination (i.e. bleeding/infection/disability), and 2 persons will die from the follow-up.

False reassurance
Even if the initial test shows no sign of the disease, it is not certain that you do not have the disease and/or will not develop it.

Discomfort:
Most people experience a discomfort of a varying intensity during the test.

Overtreatment:
Cytiolosis can be harmless and never show symptoms for some people. These individuals will therefore receive unnecessary treatment (medication and surgery). Unnecessary treatment can lead only to harm without any benefit.

[End of column 2, end of page 2]

How the screening takes place

First you are offered a test that be done at home and mailed to a laboratory where it is examined for signs of the disease.
If there is no sign of the disease, you will receive a new screening invitation in 2 years.

If your test shows sign of the disease, you will receive an offer of a follow-up examination at a hospital.
Preparation for the follow-up and the follow-up examination is experienced as unpleasant by many.
The follow-up can show if you have the disease or if you have a higher risk of developing it. It can lead to harms as listed in "Physical harms" on p. 2.
Symptoms of cytoliosis</td></tr>
</table>

	The screening programme is targeting people that has not experienced symptoms of cytoliosis yet.<br>The disease can present itself in multiple ways such as fatigue, sudden weight loss and anemia.<br><br>[End of page 3]<br><br>**What are the possible outcomes of the follow-up? There are three options:**<br>• You do not have cytoliosis or any predispositions. Your risk to develop the disease is very small.<br>• You have early signs of cytoliosis or predispositions. You will be offered treatment for this.<br>• You have cytoliosis. You will be offered an immediate and planned clinical investigation and treatment.<br><br>If you have questions about the screening programme, contact your health provider. If you show symptoms of the disease, contact your doctor. If you want to know more about benefits and harms of the screening programme, you can find more information on the internet.<br><br>[End of page 4 and the pamphlet]
Translated content pamphlet B	**An offer of a test for cytoliosis**<br>You can get yourself screened for cytoliosis, which is a non-communicable, non-hereditary disease that can be life-threatening.<br><br>***What is my risk?***<br>You reduce your risk of dying from the disease by 25% if you participate in the screening programme. **[Altered from or added to pamphlet A]**<br><br>Symptoms of the disease are known. The purpose of the test is to catch the disease before the symptoms show.<br><br>In this pamphlet, you can get a better understanding of the disease to help you decide whether to accept the offer of the test or not.<br><br>By participating in the screening programme you can reduce your risk of dying from the disease, but participation might cause harm (see p. 2 for benefits/harms).<br><br>[End of page 1]<br><br>**Potential benefits of being screened**<br><br>**Lower mortality of the disease**<br>By participating in the screening programme, you can reduce your risk of dying from the disease. If you participate, the risk of dying from cytoliosis is reduced by 25% **[Altered from or added to pamphlet A]**<br><br>**Milder treatment options**<br>If the disease is caught early there is a better chance that you can be treated by milder procedures, and your risk of going through pharmaceutical treatment is reduced.<br><br>[End of column 1, page 2]<br>**Potential harms of being screened**<br><br>**Worry and false alarm**<br>Even if the initial test shows sign of the disease, it doesn't mean that you have it – waiting for the result and the follow-up examination can lead to worry and unrest. If 10.000 persons participate in 5 consecutive screening rounds in 10 years, 3.000 persons will experience a false alarm at least once.<br><br>**Physical harm:**<br>Of the 3.000 persons who get a false alarm, 9 will experience physical harm from the follow-up examination (i.e. bleeding/infection/disability), and 2 persons will die from the follow-up.<br><br>**False reassurance**<br>Even if the initial test shows no sign of the disease, it is not certain that you do not have the disease and/or will not develop it.<br><br>**Discomfort:**<br>Most people experience a discomfort of a varying intensity during the test.<br><br>**Overtreatment:**<br>Cytiolosis can be harmless and never show symptoms for some people. These individuals will therefore receive unnecessary treatment (medication and surgery). Unnecessary treatment can lead only to harm without any benefit.<br><br>[End of column 2, end of page 2]

	**How the screening takes place**<br><br>First you are offered a test that be done at home and mailed to a laboratory where it is examined for signs of the disease.<br>If there is no sign of the disease, you will receive a new screening invitation in 2 years.<br><br>If your test shows sign of the disease, you will receive an offer of a follow-up examination at a hospital.<br>Preparation for the follow-up and the follow-up examination is experienced as unpleasant by many.<br>The follow-up can show if you have the disease or if you have a higher risk of developing it. It can lead to harms as listed in "Physical harms" on p. 2.<br>**Symptoms of cytoliosis**<br>The screening programme is targeting people that has not experienced symptoms of cytoliosis yet.<br>The disease can present itself in multiple ways such as fatigue, sudden weight loss and anemia.<br><br>[End of page 3]<br><br>**What are the possible outcomes of the follow-up? There are three options:**<br>• You do not have cytoliosis or any predispositions. Your risk to develop the disease is very small.<br>• You have early signs of cytoliosis or predispositions. You will be offered treatment for this.<br>• You have cytoliosis. You will be offered an immediate and planned clinical investigation and treatment.<br><br>If you have questions about the screening programme, contact your health provider. If you show symptoms of the disease, contact your doctor. If you want to know more about benefits and harms of the screening programme, you can find more information on the internet.<br><br>[End of page 4 and the pamphlet]
Translated content pamphlet C	**An offer of a test for cytoliosis**<br>You can get yourself screened for cytoliosis, which is a non-communicable, non-hereditary disease that can be life-threatening.<br><br>***What is my risk?***<br>1 out of 800 persons in your age-group develop cytoliosis each year. *The remaining 799 persons do not.* **[Deleted from pamphlet A]**<br><br>Symptoms of the disease are known. The purpose of the test is to catch the disease before the symptoms show.<br><br>In this pamphlet, you can get a better understanding of the disease to help you decide whether to accept the offer of the test or not.<br><u>You can reduce the risk of dying from the disease by participating in the screening programme</u>. **[Altered from or added to pamphlet A]**<br><br>**<u>Why is it important to participate?</u>**<br><u>People that have participated in the screening programme has shown to have better survival and quality of life.</u> **Altered from or added to pamphlet A]**<br><br>[End of page 1]<br><br>**Potential benefits of being screened**<br><br>**<u>Saves lives</u> [Altered from or added to pamphlet A]**<br>By participating in the screening programme, you can reduce your risk of dying from the disease – 18 out of 10.000 will die from the disease. With this screening programme 14 out of 10.000 will die from the disease. Hence, 4 out of 10.000 will avoid dying of cytoliosis.<br><br>**<u>Better treatment</u> [Altered from or added to pamphlet A]**<br>If the disease is caught early there is a better chance that you can be treated by milder procedures, and your risk of going through pharmaceutical treatment is reduced.<br><br>[End of column 1, page 2]<br>***Potential harms of being screened*** **[Deleted from pamphlet A]**<br><br>***Worry and false alarm***<br>*Even if the initial test shows sign of the disease, it doesn't mean that you have it – waiting for the result and the follow-up examination can lead to worry and unrest. If 10.000 persons participate in 5 consecutive screening rounds in 10 years, 3.000 persons will experience a false alarm at least once.* **[Deleted from pamphlet A]**<br><br>***Physical harm:***<br>*Of the 3.000 persons who get a false alarm, 9 will experience physical harm from the follow-up examination (i.e. bleeding/infection/disability), and 2 persons will die from the follow-up.* **[Deleted from pamphlet A]**

False reassurance
Even if the initial test shows no sign of the disease, it is not certain that you do not have the disease and/or will not develop it. **[Deleted from pamphlet A]**

Discomfort:
Most people experience a discomfort of a varying intensity during the test. **[Deleted from pamphlet A]**

Overtreatment:
Cytiolosis can be harmless and never show symptoms for some people. These individuals will therefore receive unnecessary treatment (medication and surgery). Unnecessary treatment can lead only to harm without any benefit. **[Deleted from pamphlet A]**

[End of column 2, end of page 2]

How the screening takes place

First you are offered a test that be done at home and mailed to a laboratory where it is examined for signs of the disease.
If there is no sign of the disease, you will receive a new screening invitation in 2 years.

If your test shows sign of the disease, you will receive an offer of a follow-up examination at a hospital.
Preparation for the follow-up and the follow-up examination is experienced as unpleasant by many. **[Deleted from pamphlet A]**
The follow-up can show if you have the disease or if you have a higher risk of developing it. The follow-up rarely leads to adverse events. **[Altered from or added to pamphlet A]**

Symptoms of cytoliosis
The screening programme is targeting people that has not experienced symptoms of cytoliosis yet.
The disease can present itself in multiple ways such as fatigue, sudden weight loss and anemia.

[End of page 3]

What are the possible outcomes of the follow-up? There are three options:

- You do not have cytoliosis or any predispositions. Your risk to develop the disease is very small.
- You have early signs of cytoliosis or predispositions. You will be offered treatment for this.
- You have cytoliosis. You will be offered an immediate and planned clinical investigation and treatment.

If you have questions about the screening programme, contact your health provider. If you show symptoms of the disease, contact your doctor. If you want to know more about benefits and harms of the screening programme, you can find more information on the internet.

[End of page 4 and the pamphlet]

Translated content pamphlet D

Call for screening of cytoliosis [Altered from or added to pamphlet A]

You have a reserved appointment for the national screening programme for a life-threatening disease. You are kindly asked to attend Bispebjerg hospital Wednesday the 2nd of August at 08.40 am. Here you will get the results of the initial home test kit (see p. 3). **[Altered from or added to pamphlet A]**

You can get yourself screened for cytoliosis, which is a non-communicable, non-hereditary disease that can be life-threatening.

What is my risk?
1 out of 800 persons in your age-group develop cytoliosis each year. The remaining 799 persons do not.

Symptoms of the disease are known. The purpose of the test is to catch the disease before the symptoms show.

In this pamphlet, you can get a better understanding of the disease to help you decide whether to accept the offer of the test or not.

By participating in the screening programme you can reduce your risk of dying from the disease, but participation might cause harm (see p. 2 for benefits/harms).

You can cancel your appointment if you call your local screening center. You will then receive a return slip. We hope you will state your reasons for cancellation so that we might optimize our future offers of cytoliosis screening. **[Altered from or added to pamphlet A**

[End of page 1]

	**Potential benefits of being screened**<br><br>**Lower mortality of the disease**<br>By participating in the screening programme, you can reduce your risk of dying from the disease – 18 out of 10.000 will die from the disease. With this screening programme 14 out of 10.000 will die from the disease. Hence, 4 out of 10.000 will avoid dying of cytoliosis.<br><br>**Milder treatment options**<br>If the disease is caught early there is a better chance that you can be treated by milder procedures, and your risk of going through pharmaceutical treatment is reduced.<br><br>[End of column 1, page 2]<br>**Potential harms of being screened**<br><br>**Worry and false alarm**<br>Even if the initial test shows sign of the disease, it doesn't mean that you have it – waiting for the result and the follow-up examination can lead to worry and unrest. If 10.000 persons participate in 5 consecutive screening rounds in 10 years, 3.000 persons will experience a false alarm at least once.<br><br>**Physical harm:**<br>Of the 3.000 persons who get a false alarm, 9 will experience physical harm from the follow-up examination (i.e. bleeding/infection/disability), and 2 persons will die from the follow-up.<br><br>**False reassurance**<br>Even if the initial test shows no sign of the disease, it is not certain that you do not have the disease and/or will not develop it.<br><br>**Discomfort:**<br>Most people experience a discomfort of a varying intensity during the test.<br><br>**Overtreatment:**<br>Cytiolosis can be harmless and never show symptoms for some people. These individuals will therefore receive unnecessary treatment (medication and surgery). Unnecessary treatment can lead only to harm without any benefit.<br><br>[End of column 2, end of page 2]<br><br>**How the screening takes place**<br><br>First you are offered a test that be done at home and mailed to a laboratory where it is examined for signs of the disease.<br>If there is no sign of the disease, you will receive a new screening invitation in 2 years.<br><br>If your test shows sign of the disease, you will receive an offer of a follow-up examination at a hospital.<br>Preparation for the follow-up and the follow-up examination is experienced as unpleasant by many.<br>The follow-up can show if you have the disease or if you have a higher risk of developing it. It can lead to harms as listed in "Physical harms" on p. 2.<br>**Symptoms of cytoliosis**<br>The screening programme is targeting people that has not experienced symptoms of cytoliosis yet.<br>The disease can present itself in multiple ways such as fatigue, sudden weight loss and anemia.<br><br>[End of page 3]<br><br>**What are the possible outcomes of the follow-up? There are three options:**<br>• You do not have cytoliosis or any predispositions. Your risk to develop the disease is very small.<br>• You have early signs of cytoliosis or predispositions. You will be offered treatment for this.<br>• You have cytoliosis. You will be offered an immediate and planned clinical investigation and treatment.<br><br>If you have questions about the screening programme, contact your health provider. If you show symptoms of the disease, contact your doctor. If you want to know more about benefits and harms of the screening programme, you can find more information on the internet.<br><br><u>We look forward to seeing you.</u> **[Altered from or added to pamphlet A]**<br><br>[End of page 4 and the pamphlet]
Translated content pamphlet E	**An offer of a test for cytoliosis**<br><br><u>We recommend that you get yourself screened for cytoliosis, which is a non-communicable, non-hereditary disease that can be life-threatening.</u> **[Altered from or added to pamphlet A**

	What is my risk?<br>1 out of 800 persons in your age-group develop cytoliosis each year. The remaining 799 persons do not.<br><br>Symptoms of the disease are known. The purpose of the test is to catch the disease before the symptoms show.<br><br>In this pamphlet, you can get a better understanding of the disease to help you decide whether to accept the offer of the test or not.<br><br>By participating in the screening programme you can reduce your risk of dying from the disease, but participation might cause harm (see p. 2 for benefits/harms).<br><br><u>The Danish Health Agency recommend participation based on a weighing of benefits and harms.</u> **[Altered from or added to pamphlet A]**<br><br>[End of page 1]<br><br>**Potential benefits of being screened**<br><br>**Lower mortality of the disease**<br>By participating in the screening programme, you can reduce your risk of dying from the disease – 18 out of 10.000 will die from the disease. With this screening programme 14 out of 10.000 will die from the disease. Hence, 4 out of 10.000 will avoid dying of cytoliosis.<br><br>**Milder treatment options**<br>If the disease is caught early there is a better chance that you can be treated by milder procedures, and your risk of going through pharmaceutical treatment is reduced.<br><br>[End of column 1, page 2]<br>**Potential harms of being screened**<br><br>**Worry and false alarm**<br>Even if the initial test shows sign of the disease, it doesn't mean that you have it – waiting for the result and the follow-up examination can lead to worry and unrest. If 10.000 persons participate in 5 consecutive screening rounds in 10 years, 3.000 persons will experience a false alarm at least once.<br><br>**Physical harm:**<br>Of the 3.000 persons who get a false alarm, 9 will experience physical harm from the follow-up examination (i.e. bleeding/infection/disability), and 2 persons will die from the follow-up.<br><br>**False reassurance**<br>Even if the initial test shows no sign of the disease, it is not certain that you do not have the disease and/or will not develop it.<br><br>**Discomfort:**<br>Most people experience a discomfort of a varying intensity during the test.<br><br>**Overtreatment:**<br>Cytiolosis can be harmless and never show symptoms for some people. These individuals will therefore receive unnecessary treatment (medication and surgery). Unnecessary treatment can lead only to harm without any benefit.<br><br>[End of column 2, end of page 2]<br><br>**How the screening takes place**<br><br>First you are offered a test that be done at home and mailed to a laboratory where it is examined for signs of the disease.<br>If there is no sign of the disease, you will receive a new screening invitation in 2 years.<br><br>If your test shows sign of the disease, you will receive an offer of a follow-up examination at a hospital.<br>Preparation for the follow-up and the follow-up examination is experienced as unpleasant by many.<br>The follow-up can show if you have the disease or if you have a higher risk of developing it. It can lead to harms as listed in "Physical harms" on p. 2.<br>**Symptoms of cytoliosis**<br>The screening programme is targeting people that has not experienced symptoms of cytoliosis yet.<br>The disease can present itself in multiple ways such as fatigue, sudden weight loss and anemia.<br><br>[End of page 3]<br><br>**What are the possible outcomes of the follow-up? There are three options:**<br>• <u>You do not have cytoliosis or any predispositions. Your risk to develop the disease is very small.</u>

	• You have early signs of cytoliosis or predispositions. You will be offered treatment for this.<br>• You have cytoliosis. You will be offered an immediate and planned clinical investigation and treatment.<br><br>If you have questions about the screening programme, contact your health provider. If you show symptoms of the disease, contact your doctor. If you want to know more about benefits and harms of the screening programme, you can find more information on the internet.<br><br>Danish and European health authorities (including the Danish Health Agency and Danish Patients) recommend participation in screening programme **[Altered from or added to pamphlet A]**<br><br>[End of page 4 and the pamphlet]
Translated content pamphlet F	An offer of a test for a life-threatening disease **[Altered from or added to pamphlet A]**<br><br>You are in risk of having the life-threatening cytoliosis that has not shown symptoms yet. Every year 5000 Danes are struck by this disease and almost 2000 die from it.<br>Many of us has experienced the disease personally through friends, family and colleagues that have fought it. You can become screened for signs of this disease. **[Altered from or added to pamphlet A]**<br>Cytoliosis is a non-communicable, non-hereditary disease that can be life-threatening.<br><br>***What is my risk?***<br>Based on your age and gender, you have increased risk of developing cytoliosis.<br><br>The symptoms of cytoliosis are vague and in many cases they show up so late that treatment has to be aggressive and might be without result. **[Altered from or added to pamphlet A]**<br>The purpose of the test is to catch the disease before the symptoms show.<br><br>In this pamphlet, you can get a better understanding of the disease to help you decide whether to accept the offer of the test or not.<br><br>By participating in the screening programme you can reduce your risk of dying from the disease, but participation might cause harm (see p. 2 for benefits/harms).<br><br>[End of page 1]<br><br>**Potential benefits of being screened**<br><br>**Lower mortality of the disease**<br>By participating in the screening programme, you can reduce your risk of dying from the disease – 18 out of 10.000 will die from the disease. With this screening programme 14 out of 10.000 will die from the disease. Hence, 4 out of 10.000 will avoid dying of cytoliosis.<br><br>**Milder treatment options**<br>If the disease is caught early there is a better chance that you can be treated by milder procedures, and your risk of going through pharmaceutical treatment is reduced.<br><br>[End of column 1, page 2]<br>**Potential harms of being screened**<br><br>**Worry and false alarm**<br>Even if the initial test shows sign of the disease, it doesn't mean that you have it – waiting for the result and the follow-up examination can lead to worry and unrest. If 10.000 persons participate in 5 consecutive screening rounds in 10 years, 3.000 persons will experience a false alarm at least once.<br><br>**Physical harm:**<br>Of the 3.000 persons who get a false alarm, 9 will experience physical harm from the follow-up examination (i.e. bleeding/infection/disability), and 2 persons will die from the follow-up.<br><br>**False reassurance**<br>Even if the initial test shows no sign of the disease, it is not certain that you do not have the disease and/or will not develop it.<br><br>**Discomfort:**<br>Most people experience a discomfort of a varying intensity during the test.<br><br>**Overtreatment:**<br>Cytiolosis can be harmless and never show symptoms for some people. These individuals will therefore receive unnecessary treatment (medication and surgery). Unnecessary treatment can lead only to harm without any benefit.<br><br>[End of column 2, end of page 2]

How the screening takes place

First you are offered a test that be done at home and mailed to a laboratory where it is examined for signs of the disease.
If there is no sign of the disease, you will receive a new screening invitation in 2 years.

If your test shows sign of the disease, you will receive an offer of a follow-up examination at a hospital.
Preparation for the follow-up and the follow-up examination is experienced as unpleasant by many.
The follow-up can show if you have the disease or if you have a higher risk of developing it. It can lead to harms as listed in “Physical harms” on p. 2.
Symptoms of cytoliosis
The screening programme is targeting people that has not experienced symptoms of cytoliosis yet.
The disease can present itself in multiple ways such as fatigue, sudden weight loss and anemia.

[End of page 3]

What are the possible outcomes of the follow-up? There are three options:

- You do not have cytoliosis or any predispositions. Your risk to develop the disease is very small.
- You have early signs of cytoliosis or predispositions. You will be offered treatment for this.
- You have cytoliosis. You will be offered an immediate and planned clinical investigation and treatment.

If you have questions about the screening programme, contact your health provider.
If you have the slightest suspicion of having the disease, it is important that you contact your doctor. **[Altered from or added to pamphlet A]**
If you want to know more about benefits and harms of the screening programme, you can find more information on the internet.

Remember, your decision about participation also affects your family and loved ones. Take the test before it is too late. **[Altered from or added to pamphlet A]**

[End of page 4 and the pamphlet]

Translated content pamphlet G

A call of a test for a life-threatening disease
You are in risk of having the life-threatening cytoliosis that has not shown symptoms yet. Every year 5000 Danes are struck by this disease and almost 2000 die from it.
You have a reserved appointment for the national screening programme for a life-threatening disease. You are kindly asked to attend Bispebjerg hospital Wednesday the 2nd of August at 08.40 am. Here you will get the results of the initial home test kit (see p. 3).
Many of us has experienced the disease personally through friends, family and colleagues that have fought it. You can become screened for signs of this disease. **[Altered from or added to pamphlet A]**
Cytoliosis is a non-communicable, non-hereditary disease that can be life-threatening.

What is my risk?
You reduce your risk of dying from the disease by 25% if you participate in the screening programme.
Therefore we recommend that you get yourself screened for signs of the disease. **[Altered from or added to pamphlet A]**

In this pamphlet, you can get a better understanding of the disease to help you decide whether to accept the offer of the test or not.

Why is it important to participate?
People that have participated in the screening programme has shown to have better survival and quality of life.

The Danish Health Agency recommend participation based on a weighing of benefits and harms. **[Altered from or added to pamphlet A]**

[End of page 1]

Potential benefits of being screened

Saves lives **[Altered from or added to pamphlet A]**
By participating in the screening programme, you can reduce your risk of dying from the disease – 18 out of 10.000 will die from the disease. With this screening programme 14 out of 10.000 will die from the disease. Hence, 4 out of 10.000 will avoid dying of cytoliosis.

Better treatment **[Altered from or added to pamphlet A]**
If the disease is caught early there is a better chance that you can be treated by milder procedures, and your risk of going through pharmaceutical treatment is reduced.

	Potential harms of being screened* [Deleted from pamphlet A]**<br><br>Worry and false alarm***<br>*Even if the initial test shows sign of the disease, it doesn't mean that you have it – waiting for the result and the follow-up examination can lead to worry and unrest. If 10.000 persons participate in 5 consecutive screening rounds in 10 years, 3.000 persons will experience a false alarm at least once.* **[Deleted from pamphlet A]**<br><br>***Physical harm:***<br>*Of the 3.000 persons who get a false alarm, 9 will experience physical harm from the follow-up examination (i.e. bleeding/infection/disability), and 2 persons will die from the follow-up.* **[Deleted from pamphlet A]**<br><br>***False reassurance***<br>*Even if the initial test shows no sign of the disease, it is not certain that you do not have the disease and/or will not develop it.* **[Deleted from pamphlet A]**<br><br>***Discomfort:***<br>*Most people experience a discomfort of a varying intensity during the test.* **[Deleted from pamphlet A]**<br><br>***Overtreatment:***<br>*Cytiolosis can be harmless and never show symptoms for some people. These individuals will therefore receive unnecessary treatment (medication and surgery). Unnecessary treatment can lead only to harm without any benefit.* **[Deleted from pamphlet A]**<br><br>[End of page 2]<br><br>**How the screening takes place**<br><br>First you are offered a test that be done at home and mailed to a laboratory where it is examined for signs of the disease.<br>If there is no sign of the disease, you will receive a new screening invitation in 2 years.<br><br>If your test shows sign of the disease, you will receive an offer of a follow-up examination at a hospital.<br>*Preparation for the follow-up and the follow-up examination is experienced as unpleasant by many.* **[Deleted from pamphlet A]**<br>The follow-up can show if you have the disease or if you have a higher risk of developing it. <u>The follow-up rarely leads to adverse events.</u> **[Altered from or added to pamphlet A]**<br><br>**Symptoms of cytoliosis**<br>The screening programme is targeting people that has not experienced symptoms of cytoliosis yet.<br>The disease can present itself in multiple ways such as fatigue, sudden weight loss and anemia.<br><br>[End of page 3]<br><br>**What are the possible outcomes of the follow-up? There are three options:**<br>• You do not have cytoliosis or any predispositions. Your risk to develop the disease is very small.<br>• You have early signs of cytoliosis or predispositions. You will be offered treatment for this.<br>• You have cytoliosis. You will be offered an immediate and planned clinical investigation and treatment.<br><br>If you have questions about the screening programme, contact your health provider.<br><u>If you have the slightest suspicion of having the disease, it is important that you contact your doctor</u>. **[Altered from or added to pamphlet A]**<br>If you want to know more about benefits and harms of the screening programme, you can find more information on the internet.<br><u>You can cancel your appointment if you call your local screening center. You will then receive a return slip. We hope you will state your reasons for cancellation so that we might optimize our future offers of cytoliosis screening. Danish and European health authorities (including the Danish Health Agency and Danish Patients) recommend participation in screening programme</u> **[Altered from or added to pamphlet A]**<br><br><u>We look forward to seeing you.</u> **[Altered from or added to pamphlet A]**<br><br>[End of page 4 and the pamphlet]
