# Supplementary material for: The impact of influences in a medical screening programme invitation: a randomized controlled trial
Source: Eur J Public Health. 2023 May 2;33(3):509–14. doi: 10.1093/eurpub/ckad067 (PMC10234657; doi:10.1093/eurpub/ckad067)
Supplement: ckad067_Supplementary_Data [file ckad067_supplementary_data.zip › ckad067_Supplementary_Data/ejph-2022-11-om-0544-File003.pdf]

APPENDIX B –INTERVIEW GUIDE TRANSLATED FROM ORIGINAL DANISH

Introduction	Hello, my name is [INTERVIEWERS NAME] and I do research at the University of Copenhagen. I am working on a study about how people react on invitations to medical screening programmes. Are you interested in helping out? It will only take you about 5 minutes.<br><br>First, I will explain how the interview will take place: We give you a pamphlet which you have to pretend is found in your mailbox. The pamphlet contains an invitation for a medical screening programme for cytoliosis which is a fictitious disease that we have come up with. It is non-transmittable and can be life-threatening. The idea is that you read this pamphlet *thoroughly* and afterwards you will answer some questions. As you read the pamphlet, please consider whether you would intend to participate in the programme if the pamphlet came in through your door. After the interview, we have a short questionnaire for you.<br><br>Now you receive the pamphlet that invites you to the medical screening programme for cytoliosis. If you have any questions, please ask.
Pamphlet	I. **Would you like to participate in this programme after having read the pamphlet?**<br><br>II. **Of these two remarks, which one do you agree with the most?**<br>1. "The pamphlet provides me information in order to help me make my own choice.<br>2. "The pamphlet provides me information in order to direct me towards the choice that the pamphlet favours."<br><br>**III. (If remark 2 is answered in II) Is there anywhere specific in the pamphlet that makes you choose this option?**
