# Supplementary material for: The impact of influences in a medical screening programme invitation: a randomized controlled trial
Source: Eur J Public Health. 2023 May 2;33(3):509–14. doi: 10.1093/eurpub/ckad067 (PMC10234657; doi:10.1093/eurpub/ckad067)
Supplement: ckad067_Supplementary_Data [file ckad067_supplementary_data.zip › ckad067_Supplementary_Data/ejph-2022-11-om-0544-File005.pdf]

# APPENDIX D – SOCIODEMOGRAPHICS OF ALL SURVEYED STRATIFIED FOR PAMPHLETS

| | All Pamphlets | Pamphlet A | Pamphlet B | Pamphlet C | Pamphlet D | Pamphlet E | Pamphlet F | Pamphlet G |
|---|---|---|---|---|---|---|---|---|
| | n (%) | n (%) | n (%) | n (%) | n (%) | n (%) | n (%) | n (%) |
| **Total** | 589 | 148 | 69 | 75 | 74 | 74 | 74 | 75 |
| **Age** | | | | | | | | |
| **≤30 years old** | 124 (21.2%) | 25 (17.4%) | 13 (18.8%) | 16 (21.3%) | 16 (21.9%) | 20 (27.0%) | 16 (21.6%) | 18 (24.0%) |
| **31-45 years old** | 180 (30.8%) | 52 (36.1%) | 27 (39.1%) | 21 (28.0%) | 26 (35.6%) | 17 (23.0%) | 22 (29.7%) | 15 (20.0%) |
| **46-60 years old** | 150 (25.7%) | 34 (23.6%) | 12 (17.4%) | 21 (28.0%) | 20 (27.4%) | 19 (25.7%) | 23 (31.1%) | 21 (28.0%) |
| **>60 years old** | 130 (22.3%) | 33 (22.9%) | 17 (24.6%) | 17 (22.7%) | 11 (15.1%) | 18 (24.3%) | 13 (17.6%) | 21 (28.0%) |
| **Missing replies - Age** | 5 | 4 | 0 | 0 | 1 | 0 | 0 | 0 |
| **Sex** | | | | | | | | |
| **Male** | 252 (43.0%) | 65 (44.8%) | 31 (44.9%) | 32 (42.7%) | 36 (48.6%) | 28 (37.8%) | 30 (40.5%) | 30 (40.0%) |
| **Female** | 334 (57.0%) | 80 (55.2%) | 38 (55.1%) | 43 (57.3%) | 38 (51.4%) | 46 (62.2%) | 44 (59.5%) | 45 (60.0%) |
| **Missing replies - Sex** | 3 | 3 | 0 | 0 | 0 | 0 | 0 | 0 |
| **Education** | | | | | | | | |
| **Primary and lower secondary education** | 35 (5.9%) | 3 (2.0%) | 6 (8.7%) | 9 (12.0%) | 5 (6.8%) | 6 (8.1%) | 3 (4.1%) | 3 (4.0%) |
| **Higher secondary education** | 63 (10.7%) | 18 (12.2%) | 7 (10.1%) | 7 (9.3%) | 10 (13.5%) | 7 (9.5%) | 8 (10.8%) | 6 (8.0%) |
| **Vocational education** | 50 (8.5%) | 10 (6.8%) | 13 (18.8%) | 6 (8.0%) | 6 (8.1%) | 6 (8.1%) | 5 (6.8%) | 4 (5.3%) |
| **Short-cycle higher education** | 50 (8.5%) | 14 (9.5%) | 3 (4.3%) | 9 (12.0%) | 4 (5.4%) | 7 (9.5%) | 10 (13.5%) | 3 (4.0%) |
| **Medium-cycle higher education** | 187 (31.7%) | 53 (35.8%) | 18 (26.1%) | 22 (29.3%) | 27 (36.5%) | 21 (28.4%) | 17 (23.0%) | 29 (38.7%) |
| **Long-cycle higher education** | 204 (34.6%) | 50 (33.8%) | 22 (31.9%) | 22 (29.3%) | 22 (29.7%) | 27 (36.5%) | 31 (41.9%) | 30 (40.0%) |
| **Missing replies - Education** | 0 | 0 | 0 | 0 | 0 | 0 | 0 | 0 |
| **Employment status** | | | | | | | | |
| **In employment** | 330 (56.0%) | 81 (54.7%) | 36 (52.2%) | 43 (57.3%) | 46 (62.2%) | 37 (50.0%) | 48 (64.9%) | 39 (52.0%) |
| **Studying** | 82 (13.9%) | 17 (11.5%) | 5 (7.2%) | 11 (14.7%) | 11 (14.9%) | 10 (13.5%) | 12 (16.2%) | 16 (21.3%) |
| **Unemployed** | 67 (11.4%) | 18 (12.2%) | 12 (17.4%) | 6 (8.0%) | 8 (10.8%) | 12 (16.2%) | 5 (6.8%) | 6 (8.0%) |
| **Retired** | 110 (18.7%) | 32 (21.6%) | 16 (23.2%) | 15 (20.0%) | 9 (12.2%) | 15 (20.3%) | 9 (12.2%) | 14 (18.7%) |
| **Missing replies – Employment status** | 0 | 0 | 0 | 0 | 0 | 0 | 0 | 0 |
| **Household status** | | | | | | | | |
| **Living alone** | 227 (38.5%) | 53 (35.8%) | 35 (50.7%) | 27 (36.0%) | 29 (39.2%) | 31 (41.9%) | 30 (40.5%) | 22 (29.3%) |
| **Living together** | 362 (61.5%) | 95 (64.2%) | 34 (49.3%) | 48 (64.0%) | 45 (60.8%) | 43 (58.1%) | 44 (59.5%) | 53 (70.7%) |

| | | | | | | | | |
|---|---|---|---|---|---|---|---|---|
| **Missing replies – Household status** | 0 | 0 | 0 | 0 | 0 | 0 | 0 | 0 |
| **Mother tongue** | | | | | | | | |
| **Danish** | 525 (89.3%) | 134 (90.5%) | 65 (94.2%) | 64 (85.3%) | 61 (82.4%) | 63 (85.1%) | 68 (93.2%) | 70 (93.3%) |
| **Other** | 63 (10.7%) | 14 (9.5%) | 4 (5.8%) | 11 (14.7%) | 13 (17.6%) | 11 (14.9%) | 5 (6.8%) | 5 (6.7%) |
| **Missing replies – Mother tongue** | 1 | 0 | 0 | 0 | 1 | 0 | 0 | 0 |
| **Location of recruitment** | | | | | | | | |
| **Herlev city centre and library** | 40 (6.8%) | 12 (8.1%) | 8 (11.6%) | 1 (1.3%) | 5 (6.8%) | 4 (5.4%) | 3 (4.1%) | 7 (9.3%) |
| **Copenhagen main library** | 40 (6.8%) | 9 (6.1%) | 3 (4.3%) | 10 (13.3%) | 2 (2.7%) | 6 (8.1%) | 5 (6.8%) | 5 (6.7%) |
| **Vanløse library and municipality centre** | 46 (7.8%) | 10 (6.8%) | 3 (4.3%) | 7 (9.3%) | 11 (14.9%) | 8 (10.8%) | 5 (6.8%) | 2 (2.7%) |
| **Øbro/Jagtvej library and municipality centre** | 29 (4.9%) | 7 (4.7%) | 5 (7.2%) | 3 (4.0%) | 1 (1.4%) | 2 (2.7%) | 8 (10.8%) | 3 (4.0%) |
| **Frederiksberg library and municipality centre** | 15 (2.5%) | 3 (2.0%) | 0 (0.0%) | 3 (4.0%) | 0 (0.0%) | 3 (4.1%) | 2 (2.7%) | 4 (5.3%) |
| **Kongens Lyngby city centre** | 15 (2.5%) | 3 (2.0%) | 1 (1.4%) | 1 (1.3%) | 2 (2.7%) | 3 (4.1%) | 3 (4.1%) | 2 (2.7%) |
| **Rentemestervej library and municipality centre** | 46 (7.8%) | 9 (6.1%) | 9 (13.0%) | 5 (6.7%) | 5 (6.8%) | 4 (5.4%) | 6 (8.1%) | 8 (10.7%) |
| **Sundby library and municipality centre** | 46 (7.8%) | 11 (7.4%) | 2 (2.9%) | 5 (6.7%) | 9 (12.2%) | 8 (10.8%) | 8 (10.8%) | 3 (4.0%) |
| **Albertslund city centre and library and municipality centre** | 15 (2.5%) | 2 (1.4%) | 7 (10.1%) | 1 (1.3%) | 2 (2.7%) | 1 (1.4%) | 2 (2.7%) | 0 (0.0%) |
| **Valby library and municipality centre** | 51 (8.7%) | 16 (10.8%) | 2 (2.9%) | 9 (12.0%) | 3 (4.1%) | 9 (12.2%) | 4 (5.4%) | 8 (10.7%) |
| **Parks (Inner Copenhagen)** | 11 (1.9%) | 4 (2.7%) | 0 (0.0%) | 0 (0.0%) | 3 (4.1%) | 2 (2.7%) | 1 (1.4%) | 1 (1.3%) |
| **Ørestad library and municipality centre** | 15 (2.5%) | 4 (2.7%) | 2 (2.9%) | 3 (4.0%) | 1 (1.4%) | 2 (2.7%) | 1 (1.4%) | 2 (2.7%) |
| **Nørrebro library and municipality centre** | 60 (10.2%) | 16 (10.8%) | 6 (8.7%) | 4 (5.3%) | 10 (13.5%) | 10 (13.5%) | 8 (10.8%) | 6 (8.0%) |
| **Frederikssund city centre** | 33 (5.6%) | 8 (5.4%) | 5 (7.2%) | 7 (9.3%) | 3 (4.1%) | 1 (1.4%) | 5 (6.8%) | 4 (5.3%) |
| **Køge city centre** | 16 (2.7%) | 3 (2.0%) | 2 (2.9%) | 2 (2.7%) | 4 (5.4%) | 2 (2.7%) | 2 (2.7%) | 1 (1.3%) |
| **Nørrebro health centre** | 12 (2.0%) | 5 (3.4%) | 1 (1.4%) | 2 (2.7%) | 1 (1.4%) | 1 (1.4%) | 0 (0.0%) | 2 (2.7%) |
| **Brønshøj library and municipality centre** | 31 (5.3%) | 10 (6.8%) | 3 (4.3%) | 5 (6.7%) | 2 (2.7%) | 2 (2.7%) | 5 (6.8%) | 4 (5.3%) |
| **Christianshavn library and municipality centre** | 6 (1.0%) | 1 (0.7%) | 2 (2.9%) | 0 (0.0%) | 0 (0.0%) | 1 (1.4%) | 1 (1.4%) | 1 (1.3%) |

| | | | | | | | | |
|---|---|---|---|---|---|---|---|---|
| **Roskilde city centre** | 62 (10.5%) | 15 (10.1%) | 8 (11.6%) | 7 (9.3%) | 10 (13.5%) | 5 (6.8%) | 5 (6.8%) | 12 (16.0%) |
| **Missing replies – Location of recruitment** | 0 | 0 | 0 | 0 | 0 | 0 | 0 | 0 |
