# Supplementary figures and images for: The impact of influences in a medical screening programme invitation: a randomized controlled trial
Source: Eur J Public Health. 2023 May 2;33(3):509–14. doi: 10.1093/eurpub/ckad067 (PMC10234657; doi:10.1093/eurpub/ckad067)

## APPENDIX C – PARTICIPANT FLOWCHART

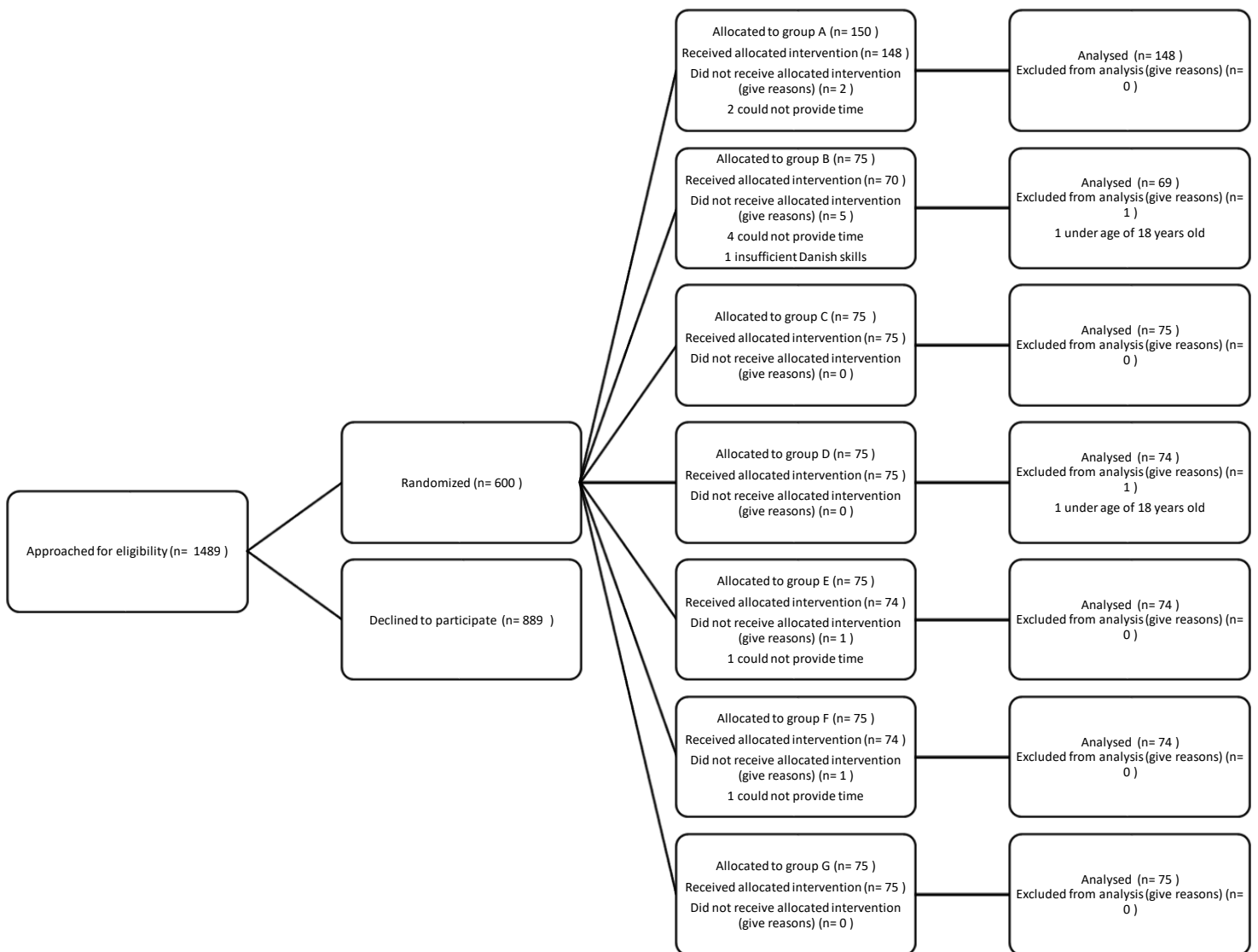

Supplement: ckad067_Supplementary_Data [file ckad067_supplementary_data.zip › ckad067_Supplementary_Data/ejph-2022-11-om-0544-File004.pdf]
